# Supplementary material for: Mixed-Method Evaluation of a Community Pharmacy Antimicrobial Stewardship Intervention (PAMSI)
Source: Healthcare (Basel). 2022 Jul 12;10(7):1288. doi: 10.3390/healthcare10071288 (PMC9323088; doi:10.3390/healthcare10071288)
Supplement: Supplementary file 1 [file healthcare-10-01288-s001.zip › healthcare-1766820-supplementary.pdf]

Supplementary File S1: Participant Questionnaires

1. PAMSI Pre-intervention questionnaire—pharmacy staff

Page 1: About you
Thank you for completing this questionnaire on your current practices around dispensing antibiotics in your community pharmacy. These questions apply to staff involved in checking the appropriateness of antibiotic prescriptions and providing infection and antibiotic information and advice to patients.

This questionnaire will take 10–15 minutes to complete.

This questionnaire will help you reflect on your current practice and help the NHS to provide tools and information to community pharmacies to help improve patient knowledge and behaviour towards their antibiotics. This reflective questionnaire can count towards your continuing professional development (CPD).

We will ask you to complete a similar questionnaire in November 2020 after using the community pharmacy Antimicrobial Stewardship Intervention (PAMSI).

Your details

1. Please provide your email address to invite you to complete the follow up questionnaire in November. We will only use this information to contact you and to match your responses. Your details will be anonymised before analysis.

Please provide the same email address in all surveys you do with us.
…………………………………………………………
If you don't have an email, give name and alternative contact:
…………………………………………………………

2. Which pharmacy do you work in?

Choose from this dropdown: [Drop down of all involved in pilot]

3. What is your role? (please tick most appropriate box):
- ☐ Pharmacist
- ☐ Pharmacy technician or ACT
- ☐ Member of dispensary team/dispenser
- ☐ Member of healthcare counter team (e.g., Healthcare assistant or healthcare partner)
- ☐ Other, please specify……………………………………………………………………

4. Have you participated in any interventions to support patients being dispensed antibiotic prescriptions in the past year since September 2019?
- ☐ Yes—please provide details……………………………….
- ☐ No
- ☐ Don't know or can't remember

5. in the past 2 years have you previously undertaken any training or continuing professional development (CPD) in antibiotic resistance, infections, or a related topic?
- ☐ No

☐ Don't know or can't remember
☐ Yes, please specify……….

6. What has your role been in dispensing antibiotics over the last 2 months? (tick all that apply)
☐ I check the antibiotic prescription for appropriateness (by this we mean you check that the antibiotic is the correct choice (if indication is known), dose, and course length for patient)
☐ I query the appropriateness of the antibiotic with the prescriber if needed
☐ I give the patient/carer antibiotic adherence/self-care advice as needed
☐ I give patient information leaflets about antibiotics and/or infections to patients
☐ None of the above
☐ Other, please specify………………….

Page 2: Your current practice, checking antibiotic appropriateness

7. Thinking about your current practice in the last 2 months, how often have you checked the appropriateness of an antibiotic prescribed? (tick one) (by this we mean you check that the antibiotic is the correct choice (if indication is known), dose, and course length for patient)
☐ Always (every antibiotic prescription)
☐ Very often (about 70% of prescriptions)
☐ Quite often (about half the prescriptions)
☐ Rarely (less than a quarter of prescriptions)
☐ Never
☐ Prefer not to say/don't know
☐ N/A

8. What prompted you to check appropriateness in the last 2 months? (tick as many as apply)
☐ It is part of my routine when checking antibiotic prescriptions
☐ If I see an unusual prescription …Q—what is unusual?
☐ If it is a prescription for repeat antibiotics
☐ If there is more than one antibiotic on a script
☐ If it is a patient in a risk group, e.g., elderly
☐ If it is for prophylaxis
☐ Use of an antibiotic checklist reminded me
☐ Other—please state
☐ Don't know or prefer not to say
☐ Not applicable or not my role

9. What, if any, were the top 3 barriers to checking appropriateness in the last 2 months? (select at least 1 and no more than 3)
☐ Insufficient knowledge of local guidance
☐ Insufficient knowledge of patient's infection
☐ Insufficient time
☐ Forgetfulness
☐ Patient not present to ask about indication/prescription
☐ High pressure situation (e.g., COVID-19)

- ☐ Insufficient easy access to patient notes
- ☐ IT issues
- ☐ There are no barriers
- ☐ Don’t know or prefer not to say
- ☐ Not applicable or not my role
- ☐ Other (please state)………………………………………………..

Page 3: Query with prescriber

10. In your current practice over the last 2 months, how often have you contacted the prescriber to query the appropriateness of an antibiotic? (tick one)

- ☐ Very often (every day)
- ☐ Often (a few times a week)
- ☐ Sometimes (a few times a month)
- ☐ Rarely (once a month)
- ☐ Never
- ☐ Don’t know or prefer not to say
- ☐ Not applicable or not my role

11. What have you queried about an antibiotic with the prescriber in the last 2 months? (tick as many as apply)

- ☐ Choice of antibiotic as it did not fit with guidelines
- ☐ Choice of antibiotic as patient allergy
- ☐ Antibiotic dose or strength
- ☐ Antibiotic course length
- ☐ Drug interaction
- ☐ Manufacture or supply issue
- ☐ Something else unusual about the antibiotic prescription
- ☐ If the antibiotic was appropriate as the patient was in a risk group, e.g., elderly
- ☐ Antibiotic prophylaxis
- ☐ Other—please state
- ☐ Prefer not to say
- ☐ Not applicable or not my role

Page 4: Your current practice, patient information, and advice

12. Thinking about your current practice in the last 2 months, how often do you provide patients with information or advice on: (tick one per row)

	Always (every patient with antibiotic prescription)	Very often (about 70% of patients with antibiotic prescriptions)	Quite often (about half the patients with antibiotic prescriptions)	Rarely (less than a quarter of patients with antibiotic prescriptions)	Never	Not applicable or not my role
Antibiotic adherence (i.e., how to take antibiotic)	☐	☐	☐	☐	☐	☐

Importance of following antibiotic adherence advice	☐	☐	☐	☐	☐	☐
Antibiotic resistance	☐	☐	☐	☐	☐	☐
Self-care advice for their infection/symptoms	☐	☐	☐	☐	☐	☐
Expected duration of their infection/symptoms	☐	☐	☐	☐	☐	☐
Signs of more serious illness—indicating they should seek further advice or help	☐	☐	☐	☐	☐	☐
Where to get further help for their infection/symptoms if needed	☐	☐	☐	☐	☐	☐

13. How often do you use the following methods to provide infection/antibiotic advice to patients?

	Always (every patient with antibiotic prescription)	Very often (about 70% of patients with antibiotic prescriptions)	Quite often (about half the patients with antibiotic prescriptions)	Rarely (less than a quarter of patients with antibiotic prescriptions)	Never	Not applicable or not my role
Speak directly to the patient	☐	☐	☐	☐	☐	☐
Provide a hard copy leaflet from official organisation (e.g., NHS or Public Health England)	☐	☐	☐	☐	☐	☐
Provide other written information	☐	☐	☐	☐	☐	☐
Direct patient to a poster or screen in the pharmacy with information	☐	☐	☐	☐	☐	☐
Signpost to a website URL link	☐	☐	☐	☐	☐	☐
Signpost to a website with QR code	☐	☐	☐	☐	☐	☐

Signpost to a website/information via a text to patient	☐	☐	☐	☐	☐	☐
Send a leaflet, website, or information by email						
Signpost to an app with further information						

Q—Any formats not listed above?

Page 5: Support needed

14. Which of the following do you think would help staff to increase their checking of antibiotic appropriateness and giving patients antibiotic/infection advice? (tick top 3)

- ☐ Training for staff on their role in this area
- ☐ A supply of patient-facing printed leaflets sent to the pharmacy covering antibiotics and infection
- ☐ Knowledge of the type of infection the patient has
- ☐ Awareness of what the patient does not understand about the antibiotic prescribed
- ☐ Awareness of what the patient does not understand about their infection
- ☐ A checklist to remind staff to check appropriateness and what advice to give
- ☐ A financial incentive
- ☐ Easy access to local antibiotic guidance
- ☐ Knowledge of patients' other illnesses and/or allergies
- ☐ Evidence on how pharmacy staff antibiotic checking or advice has made a difference to patient outcomes
- ☐ Nothing needed—have the current tools to do this
- ☐ Don’t know or prefer not to say
- ☐ Other, please state……

12a. Why do you think this?

Complete
Thank you!

2. Post-intervention questionnaire—pharmacy staff

Page 1: About you

Thank you for completing this follow up questionnaire on your current practices around dispensing antibiotics in your community pharmacy. These questions apply to staff taking part in the community Pharmacy Anti-Microbial Stewardship Intervention (PAMSI) who are involved in checking the appropriateness of antibiotic prescriptions and providing infection and antibiotic information and advice to patients.

1. Your details

Please provide the following information to help us match your response to the previous questionnaire. Your details will be anonymised before analysis.
Email: ………………………………………………………….

- ☐ Please tick if you would like to receive a certificate which can go towards CPD

2. Which pharmacy do you work in? (please select one)

[Drop down of all involved in pilot]
OR write in full
Pharmacy name_______________
Boots store number____________

3. What is your role in the community pharmacy? (please tick most appropriate box)

- ☐ Pharmacist
- ☐ Member of dispensary team
- ☐ Member of healthcare counter team
- ☐ Other, please specify………………………………………………………………

4. Have you completed any e-learning or training relating to antimicrobial stewardship in community pharmacy since taking part in PAMSI?
   - ☐ Yes, I completed the e-learning on Community pharmacy AMS on e-Learning for Healthcare website (as part of PAMSI)
   - ☐ Yes, another e-Learning not mentioned here—Q...give details
   - ☐ No, but I received training from another member of staff on PAMSI
   - ☐ No, I did not complete any e-learning and was not trained on PAMSI [why not?]

5. What has your role been in dispensing antibiotics over the last 2 months, since taking part in PAMSI? (tick all that apply)

- ☐ I have asked patient/carers to complete an Antibiotic Checklist when they hand in the prescription
- ☐ I check the antibiotic prescription for appropriateness (by this we mean you check that the antibiotic is the correct choice, dose, and course length for that patient and infection)
- ☐ I have assessed the appropriateness of the antibiotic using an Antibiotic Checklist
- ☐ I query the appropriateness of the antibiotic with the prescriber if needed
- ☐ I give the patient/carer antibiotic adherence/self-care advice as needed
- ☐ I give patient information leaflets about antibiotics and/or infections to patients
- ☐ None of the above
- ☐ Other, please specify……………….

If answer yes in q4 to checking appropriateness, go to page 2.
If do not answer yes in q4 to checking appropriateness or querying, skip.
If answer yes in q4 to checking appropriateness. go to page 2.
Skip if do not answer yes in q4 to checking appropriateness.

Page 2: Your current practice, checking antibiotic appropriateness

15. Since taking part in PAMSI over the last 2 months, how often have you checked the appropriateness of an antibiotic prescribed? (by this we mean you have checked that the antibiotic is the correct choice, dose, and course length for that patient and infection)

☐ Always (every antibiotic prescription)
☐ Very often (about 70% of prescriptions)
☐ Quite often (about half the prescriptions)
☐ Rarely (less than a quarter of prescriptions)
☐ Never
☐ Prefer not to say/don’t know
☐ N/A

16. What prompted you to check appropriateness in the last 2 months since taking part in PAMSI? (tick as many as apply or top 3)

☐ It is part of my routine when dispensing
☐ If I see an unusual prescription …Q—what is unusual?
☐ If it is a prescription for repeat antibiotics
☐ If it is a patient in a risk group, e.g., elderly
☐ If it is for prophylaxis
☐ Use of an antibiotic checklist reminded me
☐ Other—please state
☐ Don’t know or prefer not to say
☐ Not applicable or not my role

17. What, if any, were the top 3 barriers to checking appropriateness in the last 2 months since taking part in PAMSI? (select at least 1 and no more than 3)

☐ Insufficient knowledge of local guidance
☐ Insufficient knowledge of patient’s infection
☐ Insufficient time
☐ Forgetfulness
☐ High pressure situation (e.g., COVID-19)
☐ Insufficient easy access to patient notes
☐ IT issues
☐ There are no barriers
☐ Don’t know or prefer not to say
☐ Not applicable or not my role
☐ Other (please state)………………………………………………..

If said ‘query with prescriber’ in Q3

Page 3: Query with prescriber

18. Since taking part in PAMSI over the last 2 months, how often have you contacted the prescriber to query the appropriateness of an antibiotic? (tick one)

☐ Very often (every day)
☐ Often (a few times a week)
☐ Sometimes (a few times a month)

- ☐ Rarely (once a month)
- ☐ Never
- ☐ Don’t know or prefer not to say
- ☐ Not applicable or not my role

19. What have you queried about an antibiotic with the prescriber in the last 2 months, since taking part in PAMSI? (tick as many as apply)

- ☐ Choice of antibiotic as it did not fit with guidelines
- ☐ Choice of antibiotic as patient allergy
- ☐ Antibiotic dose
- ☐ Antibiotic course length
- ☐ Something else unusual about the antibiotic prescription
- ☐ If the antibiotic was appropriate as the patient was in a risk group, e.g., elderly
- ☐ Antibiotic prophylaxis
- ☐ Other—please state
- ☐ Prefer not to say
- ☐ Not applicable or not my role

If answer yes in q4 to giving self-care and adherence advice, go to page 3.
If do not answer yes in q4, skip page 3.

Page 4: Your current practice, patient information, and advice

20. Since taking part in PAMSI over the last 2 months, how often do you provide patients with information or advice on: (tick one per row)

	Always (every patient with antibiotic prescription)	Very often (about 70% of patients with antibiotic prescriptions)	Quite often (about half the patients with antibiotic prescriptions)	Rarely (less than a quarter of patients with antibiotic prescriptions)	Never	Not applicable or not my role
Antibiotic adherence (i.e., how to take antibiotic)	☐	☐	☐	☐	☐	☐
Importance of following antibiotic adherence advice	☐	☐	☐	☐	☐	☐
Antibiotic resistance	☐	☐	☐	☐	☐	☐
Self-care advice for their infection/symptoms	☐	☐	☐	☐	☐	☐
Expected duration of their infection/symptoms	☐	☐	☐	☐	☐	☐
Signs of more serious illness—indicating they should seek further advice or help	☐	☐	☐	☐	☐	☐
Where to get further help for their infection/symptoms if needed	☐	☐	☐	☐	☐	☐

21. Rank the most common ways you provide infection/antibiotic advice to patients? (where 1 is the most common way you provide advice and 9 the least common)

- ☐ Speak directly to the patient
- ☐ Provide a hard copy leaflet from official organisation (e.g., NHS or Public Health England)
- ☐ Provide other written information
- ☐ Direct patient to a poster or screen in the pharmacy with information
- ☐ Signpost to a website via a URL link
- ☐ Signpost to a website via QR code
- ☐ Signpost to a website/information via text to patient
- ☐ Send a leaflet, website or information by email
- ☐ Signpost to an app with further information

Page 5: Support needed

22. Tick the top 3 things than you feel would increase staff checking antibiotic appropriateness and patient antibiotic advice.

Select at least 1 and no more than 3.

- ☐ Nothing needed—have the current tools to do this
- ☐ A checklist to remind staff to check appropriateness and what advice to give
- ☐ Knowledge of the type of infection the patient has
- ☐ Training for staff on their role in this area
- ☐ Awareness of what the patient does not understand about the antibiotic prescribed
- ☐ Evidence on how pharmacy staff antibiotic checking or advice has made a difference to patient outcomes
- ☐ Don't know or prefer not to say
- ☐ Knowledge of patients other illnesses and/or allergies
- ☐ A supply of patient-facing printed leaflets sent to the pharmacy covering antibiotics and infection
- ☐ Easy access to local antibiotic guidance
- ☐ A financial incentive
- ☐ Awareness of what the patient does not understand about their infection
- ☐ Other, please specify

16. Please provide a reason for your answer

Page 6: Reflections compared to 2 months ago

Please say how much you agree or disagree with the following statements about yourself over the last 2 months since taking part in PAMSI, compared to the previous 2 months

	Strongly agree	Mildly agree	Neither agree or disagree	Mildly disagree	Strongly disagree	Not applicable or not my role
I am more confident to give antibiotic adherence advice to patients/carers	☐	☐	☐	☐	☐	☐
I have more opportunity to give antibiotic adherence advice to patients/carers	☐	☐	☐	☐	☐	☐
I am more motivated to give adherence advice to patients	☐	☐	☐	☐	☐	☐

when they pick up an antibiotic prescription						
I am more confident to give infection-related self-care advice to patients/carers	☐	☐	☐	☐	☐	☐
I have more opportunity to give infection-related self-care advice to patients/carers	☐	☐	☐	☐	☐	☐
I am more motivated to give self-care advice to patients when they pick up an antibiotic prescription	☐	☐	☐	☐	☐	☐
I am more confident to give infection-related safety netting advice to patients/carers	☐	☐	☐	☐	☐	☐
I have more opportunity to give infection-related safety netting advice to patients/carers	☐	☐	☐	☐	☐	☐
I am more motivated to give safety netting advice to patients when they pick up an antibiotic prescription	☐	☐	☐	☐	☐	☐
I am more confident to check the appropriateness of an antibiotic with the prescriber	☐	☐	☐	☐	☐	☐
I am more motivated to check the appropriateness of an antibiotic with the prescriber	☐	☐	☐	☐	☐	☐

Page 7: Feedback on PAMSI

13. On a typical day, with what percentage of patients/carers with an antibiotic prescription that you personally dealt with, did you use the Antibiotic Checklist with? (adherence)

(Please tick one)
0%
1–25%
26–50%
50–75%
76–99%
100%

6. Over the last 2 months, about how much extra time (if any) did it take to provide antibiotic/infection advice to each patient/carer collecting antibiotic prescriptions?

(Answer in minutes)

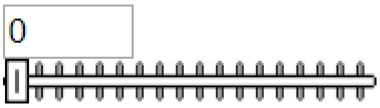

17. The time it took to complete the Antibiotic Checklist and provide tailored adherence and self-care advice to the patient is...

Please tick an option for each row, where 1 = Strongly disagree, and 5 = Strongly agree

	1 - Strongly disagree	2 - Disagree	3 - Neither agree or disagree	4 - Agree	5 - Strongly agree	N/A
feasible in every day practice	○	○	○	○	○	○
justified by the benefits to keeping antibiotics working	○	○	○	○	○	○

7. **Please add your reflections on the e-Learning, antibiotic checklist, leaflets, and other resources included in PAMSI**

(Comments box)

<u>Complete</u>

Thank you!

3. Pharmacy user questionnaire

Title—About your recent pharmacy visit to collect antibiotics
Pharmacy staff are there to help you. After your recent visit to the pharmacy to collect an antibiotic prescription, we would like to ask you about any information the pharmacy staff gave you, your experience in the pharmacy, and what you have done with the antibiotics since your visit.
This will help the pharmacy staff give you and others collecting antibiotics the best service possible.

Taking part is voluntary and will not affect your care.

We will be using information from you in order to undertake this work and will act as the data controller in compliance with GDPR. This means we are responsible for looking after your information and using it properly and keeping it safe. All your questionnaire answers will be anonymised before analysis, which means we will not know what answers are given by each person. We will not contact you for any other project, and your details will not be shared with any third parties. We will keep identifiable information about you until April 2022 at the latest.

1. **Who were the antibiotics you recently got from the pharmacy for (please tick one)?** If you picked up antibiotics for more than one person, please pick one, and answer the rest of the questions in relation to that person.
   - ☐ Myself
   - ☐ My child
   - ☐ A relative
   - ☐ Prefer not to say
   - ☐ Other, please specify….

2. **Which pharmacy did you get the antibiotics from? (please select one)**
   Town and city:
   Pharmacy name:
   [drop down of the pharmacies in pilot]

If the antibiotics were not for you, please fill in the rest of the questionnaire for the person named on the prescription.

3. **What antibiotics have you been prescribed for your recent infection (please tick one)?** If you picked up more than one antibiotic, please select one, and answer the rest of the questions in relation to that antibiotic.

[Drop-down list]

- ☐ Amoxicillin
- ☐ Flucloxacillin
- ☐ Nitrofurantoin
- ☐ Doxycycline
- ☐ Clarithromycin
- ☐ Ciprofloxacin
- ☐ Phenoxymethylpenicillin (Penicillin V)
- ☐ Metronidazole
- ☐ Trimethoprim

- ☐ Co-Amoxiclav
- ☐ Other, please specify
- ☐ I don’t know/remember
- ☐ Prefer not to say

Your visit to the pharmacy
Please answer these questions about your visit to the pharmacy to pick up the antibiotic prescription.

4. **Which of the following information about antibiotics or infections did you receive during your visit to collect antibiotics? (tick all that apply)**
   - ☐ When to take the antibiotics
   - ☐ How long to take the antibiotics
   - ☐ When to take the antibiotics around food
   - ☐ If I can drink alcohol whilst taking the antibiotics
   - ☐ Possible side effects from the antibiotics
   - ☐ When to seek further help for the infection
   - ☐ How long it would take to feel better from the infection
   - ☐ Actions I could take to help myself feel better (e.g., self-care treatment)
   - ☐ What to do with any unused antibiotics
   - ☐ Antibiotic resistance
   - ☐ The pharmacy staff did not give me any advice about antibiotics or infections
   - ☐ I don’t remember the advice they provided
   - ☐ They provided advice on something not stated here….Q—Please state
   - ☐ Prefer not to say

5. **Following your visit to the pharmacist to pick up the antibiotic prescription—please say how much you agree or disagree with the following statements:**

	Strongly agree	Mildly agree	Neither agree or disagree	Mildly disagree	Strongly disagree	Not applicable
I now feel more able to look after my infection	☐	☐	☐	☐	☐	☐
I know how to take my antibiotics	☐	☐	☐	☐	☐	☐
I know how long my infection will probably last	☐	☐	☐	☐	☐	☐
I know **when** I need to seek further help with my infection	☐	☐	☐	☐	☐	☐
I know **where** I should seek further help for my infection if I need it	☐	☐	☐	☐	☐	☐
**I know more about how to help prevent another infection**	☐	☐	☐	☐	☐	☐

How you took your antibiotics

6. **Did you finish the course of antibiotics you collected? (please tick one)**

- ☐ Yes I took all of it
- ☐ Yes most of it
- ☐ Yes some of it
- ☐ No none of it
- ☐ Can’t remember
- ☐ Prefer not to say

7. For the majority of the time you were taking antibiotics, how did you take the medicine? (tick all that apply)

- ☐ At regular intervals throughout the day
- ☐ As my doctor/nurse/pharmacist advised
- ☐ All at once each day
- ☐ Whenever I remembered to
- ☐ I would rather not say
- ☐ Other (please specify)

8. Did you know whether your antibiotic needed to be taken with food, and did you follow this advice? (tick one)

- ☐ I was given specific instructions for food and I followed these
- ☐ I was given specific instructions for food but did not follow these
- ☐ I was not given any specific instructions about taking my antibiotic with food
- ☐ I was told it did not matter when I took the antibiotic in relation to food
- ☐ I would prefer not to say
- ☐ I can’t remember/don’t know

9. Did you know whether you could drink alcohol while taking your antibiotic and did you follow this advice? (tick one)

- ☐ I was told not to drink alcohol while on these antibiotics, and I did not drink alcohol
- ☐ I was told not to drink alcohol while on these antibiotics, but I did not follow this
- ☐ I was told it was fine to drink alcohol while on these antibiotics
- ☐ I was not given any specific instructions about drinking alcohol while on these antibiotics
- ☐ I would prefer not to say
- ☐ I can’t remember

Page 5

Answer: Space the doses evenly throughout the day, as directed on the packet or the patient information leaflet that comes with the medication, or as instructed by your GP, nurse, or pharmacist. Keep taking this medicine until the course is finished, unless you are told to stop.

It’s essential to take antibiotics as prescribed by your healthcare professional.
Visit https://www.nhs.uk/conditions/antibiotics/ for what to do if you miss a dose of antibiotics or accidentally take an extra dose.

You are allowed to drink alcohol with most antibiotics. The exceptions are: metronidazole (you should not drink alcohol during the course and for 48 hours after treatment) and tinidazole (you should not drink alcohol during the course and for 72 hours after treatment).

[If answer yes most of it, yes some of it, no none of it to Q7]

10. Why did you not take all of the antibiotics? (tick all that apply)

- ☐ I started to feel better
- ☐ The antibiotics made me feel poorly
- ☐ My doctor/nurse/pharmacist advised me to stop
- ☐ I had side effects
- ☐ Other (please specify)
- ☐ I'd prefer not to say
- ☐ I can't remember/I don't know

[If answer yes most of it, yes some of it, no none of it to Q7]

11. If you stopped taking the antibiotics, at what day in the course did you stop? (open)

12. If you had unused antibiotics, what did you do with them? (tick one)

- ☐ I returned them to a pharmacy
- ☐ I flushed them down the sink or toilet
- ☐ I put them in the bin
- ☐ I shared them with family or friends
- ☐ I gave them to my pet
- ☐ I still have them
- ☐ Other (Please specify)
- ☐ I do not have any unused antibiotics
- ☐ I'd prefer not to say
- ☐ I can't remember/I don't know

13. [If still have them] **Why do you still have your unused antibiotics? (tick all that apply)**

- ☐ To save them in case I get ill in the future
- ☐ To save them in case family or friends get ill in the future
- ☐ To save them in case a pet gets ill in the future
- ☐ I intend to dispose of them but haven't had a chance to
- ☐ I intend to take them back to a pharmacy, but haven't had the chance to
- ☐ Other (please specify)

ANSWER: Pharmacies can take back unused medication to make sure that they are safely disposed of. Antibiotics flushed down the toilet get into our water supply. You should not share antibiotics with other people or pets, as antibiotics are specific for each infection and person. Using antibiotics kills some of our gut bacteria and increases antibiotic resistance in other gut microbes.

14. Thinking of the illness you received the antibiotics for, which of the following actions, if any, did you take as a result after picking up the antibiotic prescription?

- ☐ I read the leaflet the pharmacy staff gave me
- ☐ I visited the website the pharmacy staff gave me the details of
- ☐ I contacted or visited my local doctor's surgery to talk to a GP or nurse

☐ I asked for advice at the same pharmacy I picked up my prescription from
☐ I asked for advice at a different pharmacy, not where I got the prescription
☐ I telephoned NHS 111 or other out of hours service
☐ I used the NHS website
☐ I used other health websites (e.g., patient.co.uk, webMD)
☐ I visited an NHS walk-in centre/minor urgent care centre/minor injuries centre
☐ I visited a GP out of hours service
☐ I visited a hospital accident and emergency (A&E)
☐ I got advice from a friend/family member/colleague
☐ I used social media for advice (e.g., Facebook, Twitter, Pinterest, Instagram)
☐ I don't remember
Other (please specify)

Flu vaccine

15. At your recent pharmacy visit to pick up an antibiotic prescription, what actions if any happened around the flu vaccine? (tick all that apply)

☐ I was given advice about **if** I needed the flu vaccine
☐ I was given advice about why I needed the flu vaccine
☐ I was given a leaflet about the flu vaccine
☐ I was offered a flu vaccine
☐ I made an appointment to have a flu vaccine
☐ I was not given any advice about the flu vaccine
☐ I can't remember or prefer not to say
☐ I had already received the flu vaccine

16. Following your pharmacy visit, did you get your flu vaccine? (tick one)

☐ I had already had my flu vaccine
☐ I am not eligible for the flu vaccine
☐ Yes, I have now had my flu vaccine
☐ I still intend to, but have not yet had my flu vaccine
☐ I do not intend to get the flu vaccine
☐ Other, please specify
☐ Prefer not to say

The flu vaccine is routinely given on the NHS to:

- adults 65 and over
- people with certain medical conditions (including children in at-risk groups from 6 months of age)
- pregnant women
- children aged 2 and 3
- children in reception class and school years 1, 2, 3, 4, and 5

For more information about the flu vaccine, speak to your GP, practice nurse, or pharmacist, or visit: https://www.nhs.uk/conditions/vaccinations/flu-influenza-vaccine/

About you
Please answer the following questions about you to help us better understand how to help patients who visit the pharmacy.

17. Which of the following best describes how you think of yourself?

- ☐ Male
- ☐ Female
- ☐ In another way
- ☐ Prefer not to say

18. Which age group applies to you?

- ☐ **Under 16**
- ☐ 16–24
- ☐ 25–34
- ☐ 35–44
- ☐ 45–54
- ☐ 55–59
- ☐ 60–64
- ☐ 65–74
- ☐ 75+

19. Which, if any of the following apply to you? I am the parent or legal guardian of...

A child/children under the age of 16 who live/s with me
A child/children aged under 16 who does not/do not live with me and to whose upbringing I contribute financially
A child/children aged under 16 who does not/do not live with me and to whose upbringing I do not contribute financially
A child/children aged 16 to 19
None of these

20. Which ethnic group do you consider you belong to?

White—English/Welsh/Scottish/Northern Irish/British
White—Irish
White—Gypsy or Irish Traveller
White—Any other White background
Mixed—White and Black Caribbean
Mixed—White and Black African
Mixed—White and Asian
Mixed—Any other Mixed/multiple ethnic background
Asian/Asian British—Indian
Asian/Asian British—Pakistani
Asian/Asian British—Bangladeshi
Asian/Asian British—Chinese
Asian/Asian British—Any other Asian background
Black—African
Black—Caribbean
Black—Any other Black/African/Caribbean background
Arab
Any other ethnic group
Do not know
Prefer not to say

Thank you for taking the time to answer these questions. Your answers will be vital to help the pharmacy team help you in the best way they can.

Supplementary File S2: Pharmacy Staff interview schedule

Community Pharmacy AMS intervention: Pharmacist interviews

Introductions

- This discussion will take approx. 45 min to 1 h.
- This is a discussion to explore experiences of using the TARGET materials during the trial and how they worked in a COVID-19 context.
- Everything discussed will be kept confidential.
- Any personal information disclosed will be anonymised.
- This is a discussion with no right or wrong answers.
- To tell you a little bit about the structure—we will start with how you found using the materials, the roles of different staff, and how it was during COVID-19. Then we will open it up for anything you yourself would like to add.

Taking part

1. **How did you find it?**

Prompts

- How did you find it?
- Did you use all the materials?

In the toolkit there was checklist, leaflets, and the e-learning so we can go through each of these in more detail.
Dependent if they used all of them.

2. How did you find using the checklist

Prompts

- Talk through how you used it with each patient.
- How did you remember how to use it?
- Which staff where involved/who else used tools?
    - Did you have to train other staff or was it self-explanatory?
- Did patients always complete or did staff sometimes on their behalf?
- How did you approach using the checklist with deliveries/proxy visitors?
- How do you believe the checklist has affected the advice/information you give to patients, if at all?

3. **Did you use the patient information leaflets and how did you find those?**

Prompts

- How often did you use them?
- Do you as a pharmacy do deliveries? Did you use leaflets with these?
- How were the patient information leaflets received by the patients?

- We have a new leaflet regarding self-care of infections. Did you use it? Did you find it useful?

4. Did you do the E-learning and how did you find it?

Prompts

- Did you learn anything? Were you surprised by anything?

- Difference between those pharmacists who used e-learning those who didn't (engagement).

Impact

5. Did taking part in this pilot have any impact on you and other staff, if at all?

Prompts

- Did you change your approach?
- Thought into the process.

6. Did you notice as a team there was a change in your practise?

Prompts

- Approach to your role/responsibilities

To reiterate, there is no expectation

7. Would you use the TARGET tools again? Would you welcome it into regular practise?

Barriers to using the materials

8. What if anything prevented you from using the printed materials?

Prompts

- Time?
- Delivering prescriptions?
- Flu season (Did you find a difference in the use of the checklist during different times?)

9. Were there any groups of patients that you felt the intervention was not suitable for?

Prompts

- e.g., Learning disabilities, language barriers

10. In the post questionnaire, the average additional minutes to normal practise when using the checklist was 5 minutes—does this sound right?

Prompts

- Was this extra time feasible to incorporate in normal practise?

11. Is there any information you think is not included or could be added to the checklist or other materials?

Supplementary 3: Patient/carer follow up questionnaire findings

Pharmacy staff used the TARGET Antibiotic Checklists with 2043 patients over the data collection period; 884 patients provided contact details, and 89 (10%) responded to a follow up questionnaire after their pharmacy visit. See Table S1 for demographic information. Patients recalled receiving information from pharmacy staff on how long to take antibiotics (75%), information regarding food consumption (72%), alcohol consumption (44%), side effects (44%), and how long it would take to feel better (30%).

Patients' self-reported knowledge of managing their infection post-pharmacy visit was high (Figure S1), particularly on how to take their antibiotics and awareness of seeking further help for their infection. A total of 86/89 (97%) reported adherence to their antibiotic regime; one patient did not start antibiotics, and two stopped without advice from a healthcare professional (HCP) (due to side effects or feeling better).

Table S1: Self-reported demographic data of patients/carers who participated in follow up questionnaire.

Patients' characteristics (n = 89)	Number (%)
**Gender**	
Female	62 (70%)
Male	27 (30%)
**Age**	
Under 16	2 (2%)
16–34	23 (26%)
35–54	31 (35%)
55–74	25 (28%)
75+	5 (6%)
Missing	3 (3%)
**Ethnicity**	
White—English/Welsh/Scottish/Northern Irish/British	69 (78%)
White—Irish	1 (1%)
White—any other background	11 (12%)
Mixed—White and Black African	1 (1%)
Mixed—Any other Mixed/multiple ethnic background	1 (1%)
Asian/Asian British	1 (1%)
Black—African	1 (1%)
Any other ethnic group	2 (2%)
Prefer not to say	1 (1%)
Missing	1 (1%)

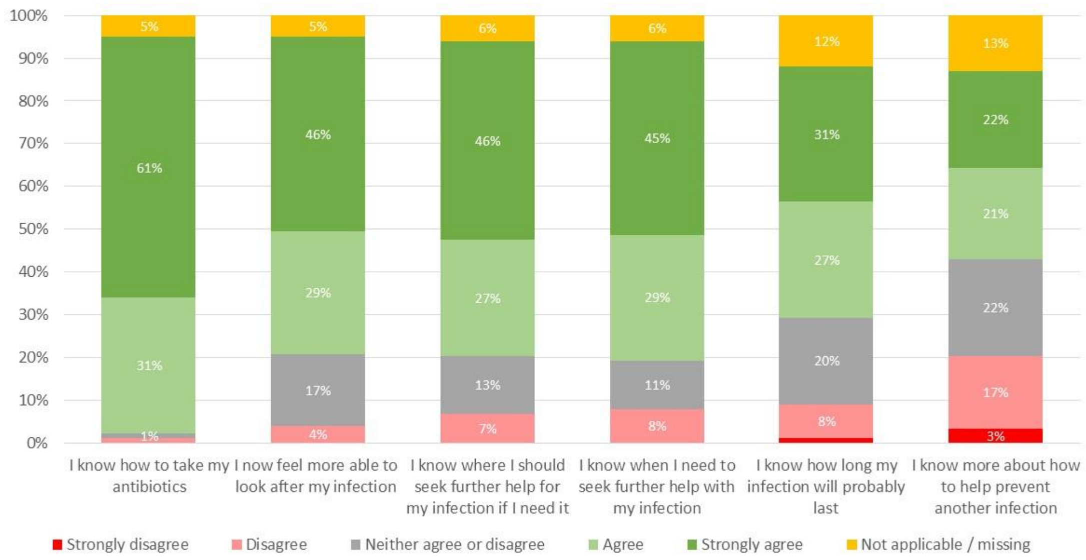

Figure S1: Patient/carers' (n=89) knowledge reported after their pharmacy visit, when followed up weekly by the research team with a questionnaire.
